# Kinematics and Flow Field Analysis of *Allomyrina dichotoma* Flight

**DOI:** 10.3390/biomimetics9120777

**Published:** 2024-12-20

**Authors:** Huan Shen, Kai Cao, Chao Liu, Zhiyuan Mao, Qian Li, Qingfei Han, Yi Sun, Zhikang Yang, Youzhi Xu, Shutao Wu, Jiajun Xu, Aihong Ji

**Affiliations:** 1 Lab of Locomotion Bioinspiration and Intelligent Robots, College of Mechanical and Electrical Engineering, Nanjing University of Aeronautics and Astronautics, Nanjing 210016, China; shenhuan99@nuaa.edu.cn (H.S.); caokai@nuaa.edu.cn (K.C.); mao1010051495@163.com (Z.M.); hanqf@nuaa.edu.cn (Q.H.); nuaa_sunyi@nuaa.edu.cn (Y.S.); zhikangyang@nuaa.edu.cn (Z.Y.); xuyouhzi@nuaa.edu.cn (Y.X.); wushutao522@nuaa.edu.cn (S.W.); xujiajun@nuaa.edu.cn (J.X.); 2 School of Mechanical and Electrical Engineering, Soochow University, No. 8, Jixue Road, Suzhou 215131, China; c.liu@suda.edu.cn; 3 College of Mechanical and Electrical Engineering, China Jiliang University, 258 Xueyuan Street, Hangzhou 310018, China; qianli881@nuaa.edu.cn

**Keywords:** *Allomyrina dichotoma* flight, wing deployment, flapping kinematics, flow field visualization, computational fluid dynamics

## Abstract

In recent years, bioinspired insect flight has become a prominent research area, with a particular focus on beetle-inspired aerial vehicles. Studying the unique flight mechanisms and structural characteristics of beetles has significant implications for the optimization of biomimetic flying devices. Among beetles, *Allomyrina dichotoma* (rhinoceros beetle) exhibits a distinct wing deployment–flight–retraction sequence, whereby the interaction between the hindwings and protective elytra contributes to lift generation and maintenance. This study investigates *A. dichotoma’s* wing deployment, flight, and retraction behaviors through motion analysis, uncovering the critical role of the elytra in wing folding. We capture the kinematic parameters throughout the entire flight process and develop an accurate kinematic model of *A. dichotoma* flight. Using smoke visualization, we analyze the flow field generated during flight, revealing the formation of enhanced leading-edge vortices and attached vortices during both upstroke and downstroke phases. These findings uncover the high-lift mechanism underlying *A. dichotoma*’s flight dynamics, offering valuable insights for optimizing beetle-inspired micro aerial vehicles.

## 1. Introduction

With the rapid advancement of micro aerial vehicle technology, there has been growing interest in bioinspired design within the engineering community [1,2]. Insect flight, known for its exceptional maneuverability and aerodynamic efficiency, has become a key source of inspiration for the design of such vehicles [3]. By studying the detailed wing kinematics and flow field characteristics of insects, valuable insights can be gained for optimizing the structural design and aerodynamic performance of new flying devices [4,5]. In recent years, advancements in computation, flow visualization, and robotics have led to breakthroughs in understanding insect flight aerodynamics both domestically and internationally [6,7]. Computational Fluid Dynamics (CFD) enables the development of numerical models for insect flight [8,9], while high-speed flow visualization and Particle Image Velocimetry (PIV) provide quantitative measurements of the insects and their surrounding flow fields [10,11]. Furthermore, robotic models, dynamically scaled to the actual size of insects, offer physical representations of butterfly flight [12]. These technological advancements present practical and feasible methods for exploring the underlying mechanisms of insect flight.

The agile and highly maneuverable flight of insects is achieved through their complex flapping-wing motions. By using high-speed imaging technology to capture the wing movements of insects, researchers can obtain various parameters that serve as the foundation for analyzing insect flight behavior. Muijres et al. [13] measured the kinematic parameters of the body and wings during the flight of fruit flies, investigating the rapid turning mechanism during their escape maneuvers. Nguyen et al. [14] studied the flight parameters of *Allomyrina dichotoma*, analyzing the relationship between its body posture and wingbeat dynamics. Sun Mao and colleagues systematically explored the high-lift and flexible control mechanisms of fruit flies during classical flight states [15]. Farisenkov conducted research on the flight of rove beetles [16], discovering that the unique movement pattern of their fringed wings grants them exceptional flight capabilities.

Due to the inherent instability of the surrounding flow field during insect flight, analyzing wing motion alone is insufficient for a comprehensive understanding. Visualizing the flow field during flight is essential to uncover the mechanisms behind the remarkable aerodynamic performance of flying insects. Thomas [17] observed the wake vortices generated by butterflies and studied the vortex structure to investigate the wake capture mechanism. Bomphrey [18] analyzed the topological structure of the wake vortices of freely flying bumblebees, focusing on the leading-edge vortex attachment mechanism. Nakata [8] examined the free flight of mosquitoes and found that their long, slender wings generate sufficient aerodynamic force through a combination of wake capture and rotational drag mechanisms. The aforementioned unsteady aerodynamic mechanisms partially explain the generation of high lift during insect flight, with the most crucial factor being the evolution of the leading-edge vortex during the wing flapping process [12].

The current research on beetle flight mechanics primarily focuses on their kinematics, inferring the underlying aerodynamic mechanisms through the analysis of wing motion during free flight or takeoff processes, while lacking detailed capture and analysis of the actual flow field [14,19]. It is well-known that the forewings of beetles have evolved into hardened elytra, which mainly serve to protect the softer hindwings. As relatively large insects, beetles are capable of overcoming gravity during flight by relying solely on the motion of their hindwings. The specific evolution of the leading-edge vortex generated by the flapping motion of the hindwings is crucial for understanding their aerodynamic mechanisms [20,21]. On the other hand, the role of the elytra in flight is also significant and should not be overlooked. In confined spaces or unfavorable environments, the hindwings are folded and stored beneath the elytra. When encountering predators or while foraging, the elytra are released, and the hindwings expand. The mechanism behind this unique wing deployment/retraction behavior has attracted the attention of researchers. For example, Hass and Beutel studied the wing deployment process of *Potosia brevitarsis*, proposing that the mechanism is powered by abdominal and thoracic muscles [22]. Leoni’s research on the autonomous and induced motion of the tethered beetle suggests that the lifting motion of the prothorax contributes to the closure of the elytra [23]. During flight, the elytra may also interact with the vortices generated by the hindwings. The flow field changes caused by the wing–wing interaction may further contribute to the generation of lift [21,24]. This study focuses on *A*. *dichotoma* and analyzes its wing deployment/retraction process, wing motion trajectory, and vortices generated during wing motion. These findings reveal the intriguing flight mechanisms of the beetle, providing valuable insights for the structural design and aerodynamic optimization of biomimetic aerial vehicles.

## 2. Materials and Methods

### 2.1. Specimens

**Figure 1:**
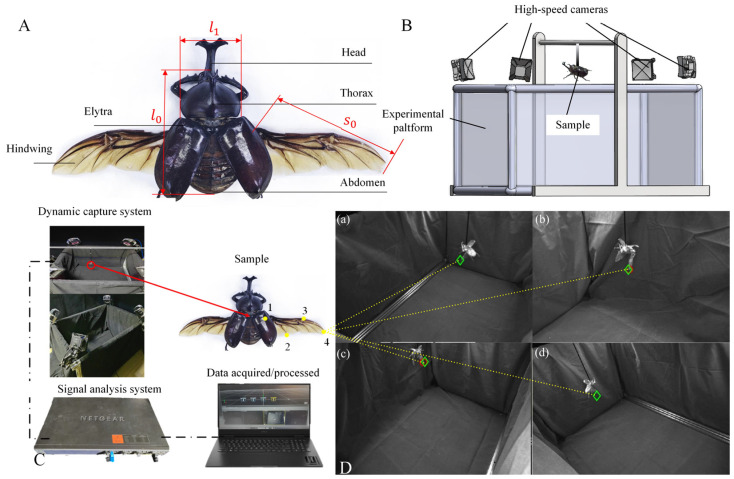
Setup of the kinematic experiment. (**A**) Schematic of macroscopic morphological parameters. (**B**) Schematic of the experimental setup. (**C**) Specific components of the motion capture system. (**D**) Marked points clearly captured by the four high-speed cameras.

*A. dichotoma* belongs to the order *Coleoptera* and the family *Scarabaeidae*, with its body structure illustrated in Figure 1. Adult *A. dichotoma* exhibit high activity levels at night during the summer months. Consequently, insect kinematic experiments were conducted during the summer evenings. Specimens were captured at the foothills of Zijin Mountain in Nanjing, Jiangsu Province (118.80° E, 32.10° N), and were maintained in a laboratory under humid conditions (temperature 25 °C–27 °C, humidity 65–70%). The beetles were fed decaying fruits daily to ensure their normal activity levels with a simulated 12 h light/12 h dark cycle. After a two-day acclimation period, during which the beetles became familiar with the laboratory environment, the flight observation experiments commenced. Upon completion of the experiments, morphological measurements of the specimens were conducted, with results summarized in Table 1. Here, *M*_1_, *M*_2_, and *M*_3_ represent the total body mass, hindwing mass, and elytron mass of *A. dichotoma*, respectively. Additionally, *s*_0_ and *s*_1_ denote the span length and mean chord length of the hindwings, *l*_0_ represents body length, *l*_1_ indicates body width, *s*_0_/*s*_1_ signifies the aspect ratio of the hindwings, *l*_0_/*l*_1_ refers to the body length-to-width ratio, *A*_w_ represents the surface area of the hindwings, and *γ* denotes the surface mass density of the hindwings.

### 2.2. Motion Capture and Motion Measurement

To obtain the wing movement characteristics of *A. dichotoma* in the tethered state, a motion measurement system was established. As shown in Figure 1C, this system consists of four OptiTrack high-speed cameras (Prime 17W, NaturalPoint, Inc., Corvallis, OR, USA), an experimental platform, and a computer system. The experimental platform measures 90 cm in length and 50 cm in width, while the fixed height of the camera rig is set at 50 cm. The four cameras are arranged in a quadrilateral formation, securely mounted on the rig to surround the insect under investigation, as shown in Figure 1B. To identify the center of mass, we attached a thread to *A. dichotoma*. Once the beetle came to rest, its center of mass is located under the external point from which it was hanging. Because we had already determined the direction of gravity, we could thus determine a line which goes through the center of mass, relative to its attached body frame. We did not actively stimulate the *A. dichotoma* to fly but instead allowed the beetles to voluntarily unfold their wings. Based on the wingbeat frequency of the beetles, the high-speed cameras were configured to capture in post-trigger mode at a frame rate of 360 frames per second (fps) with a shutter speed of 2000 fps, and imaging was conducted at a resolution of 1664 × 1088 pixels. The aperture and focal length of the cameras were adjusted to ensure that both the body and wings of the *A. dichotoma* were clearly visible in the high-speed recordings [25,26,27].

The dorsal part of the mesothorax of *A. dichotoma* was secured to a cylindrical carbon rod with a diameter of 3 mm using α-cyanoacrylate adhesive. The carbon rod was then fixed to the rigid framework of the experimental platform to minimize vibrations caused by the insect’s movements. When the beetle deployed its hindwings, four reference points were marked using yellow acrylic paint: the wing root (Point 1), midsection of the trailing edge (Point 2), midsection of the leading edge (Point 3), and the wingtip (Point 4), as illustrated in Figure 1C. At the moment the beetle flapped its wings, the cameras were triggered synchronously to capture the flapping motion of *A. dichotoma*. The three-dimensional coordinates of the beetle’s flight movements at any instant were then obtained using Direct Linear Transformation (DLT) methodology [28,29]. The experiment involved five adult *A. dichotoma* beetles (three males and two females), from which a total of 20 complete wing unfolding/folding trials and 25 wingbeat flight trials were conducted. Each flight trial captured more than 10 complete wingbeat cycles.

### 2.3. Calibration and Data Processing

To describe the flight speed and trajectory of *A. dichotoma’s* wings, the coordinates obtained using the DLT method were used as the inertial reference frame. The inertial coordinate system, *N*_g_, was fixed relative to the position of the experimental platform, while the body-fixed coordinate system, *N*_b_, had its origin at the beetle’s center of mass. The X_b_ axes were defined such that one axis pointed from the right wing to the left wing, and Y_b_ extended from the beetle’s tail to its head. Since the body of *A. dichotoma* is fixed, the body coordinate system *N*_b_ can be set to coincide with the inertial coordinate system *N*_g_.

In the stroke plane, the wing coordinate system $N_{w}=\{x_{w},y_{w},z_{w}\}$ is defined by $x_{w}$ along the leading edge of the wing, $y_{w}$ parallel to the chord perpendicular to the leading edge, and $z_{w}$ perpendicular to the $x_{w}$–$y_{w}$ plane. The relationship between the vector $v_{b}^{F_{b}}$ in the body coordinate system and the vector $v_{b}^{F_{w}}$ in the wing coordinate system is displayed as

(1) $$v_{b}^{F_{w}}=R_{b}^{w}\cdot v_{b}^{F_{b}}$$

**Figure 2:**
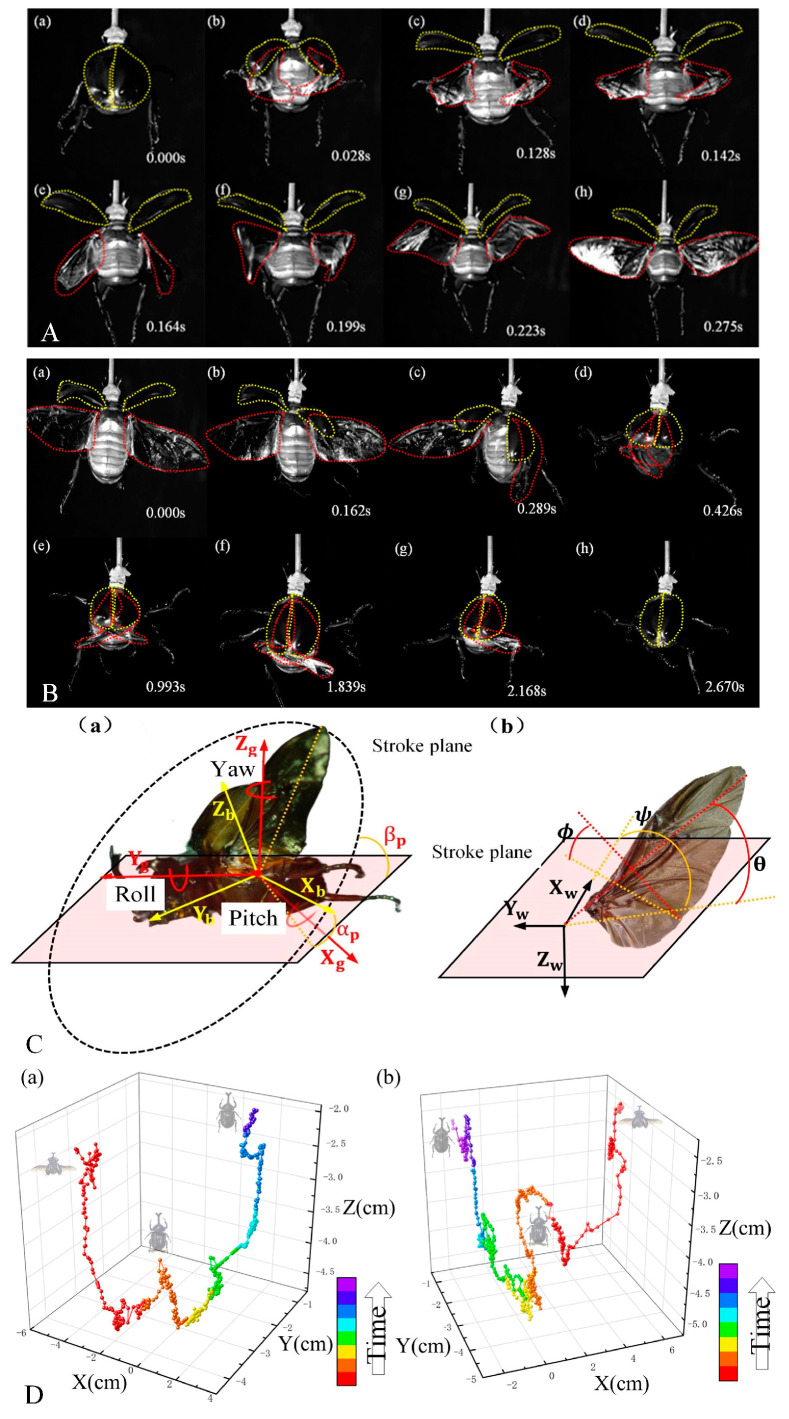
Wing extension/folding process and quantified time sequence of *A. dichotoma*. (**A**) Wing extension process of *A. dichotoma*. (**B**) Wing-folding process of *A. dichotoma*. The red dashed lines represent the hindwing outline and the yellow dashed lines represent the elytra outline. (**C**) Reference frame transformations and parameter definitions during the flight of *A. dichotoma*. (**a**) Relationship between the inertial coordinate system and body coordinate system. (**b**) Relationship between the body coordinate system and wing coordinate system. (**D**) Wingtip trajectory during wing folding. (**a**) Left wingtip trajectory. (**b**) Right wingtip trajectory.

The orientation of the stroke plane is described relative to the body frame of reference using two angles, α_p_, and β_p_, which represent the rotation of the stroke plane about the body z-axis, and the inclination of the stroke plane. These angles, α_p_, and β_p_, are solved for simultaneously by varying them to minimize the square of the angle between the pitching axis and stroke plane summed over the entire flight. This was performed for the left and right wings simultaneously so that any asymmetries in the data arose from the wing kinematics, not from differences in the stroke plane orientations, as shown in Figure 2C. We then have

(2) $$R_{b}^{w}=R_{b}^{s}\cdot R_{s}^{w}$$

$R_{b}^{s}$ rotates $N_{b}$ to the stroke plane. The flapping angle ψ, torsion angle ϕ, and swing angle θ of wing movement are then obtained. The first rotation, flapping angle ψ, is the stroke angle of the wing. This angle represents the flapping motion of the wing along the stroke plane and is positive when the wing is above the x–y plane. The second rotation, swing angle θ, is the deviation angle of the wing, representing how far away from the stroke plane the pitching axis is. This is the value which was minimized when determining the pitching axis and stroke plane orientation, and thus is typically small compared to the other two angles. The last rotation, torsion angle ϕ, is the pitching angle, which is how much the wing has rotated about its pitching axis. As shown in Figure 2C, by transforming between these coordinate systems, the wing motion of *A. dichotoma* can be more accurately defined, with detailed variations described in reference [30,31].

### 2.4. Flow Field Visualization Experiment and CFD Verification

To further investigate the aerodynamic characteristics of *A. dichotoma* in flight, a smoke visualization technique was employed to capture the flow field around the beetle’s body. The visualization system consisted of a custom-built micro wind tunnel, a high-speed camera (VEO 1310s, Phantom, NC, USA), a smoke generator, and a light source. The wind tunnel was of an open-circuit type, with dimensions of 600 × 500 × 400 mm (streamwise, vertical, and spanwise directions), and the test section had circular inlet and outlet diameters of 120 mm. The airflow speed could be adjusted from 0 to 15 m/s, with 1.2 m/s selected in this experiment to match the free-flight speed of the beetle [32]. A Zeiss 50 mm macro lens was used for the high-speed camera, set to a resolution of 2560 × 1600 pixels and a frame rate of 6000 fps, which allowed the clear capture of smoke flow details around the hind wings. The smoke generator uses a mixture of glycerin and propylene glycol to produce smoke. An LED light source was used to uniformly illuminate the smoke lines, providing high-intensity scattered light, ensuring that the smoke layers appeared clearly in the high-speed camera footage.

The flight process of the beetle was simulated using the commercial CFD software XFlow 2019x (Next Limit Technologies, Madrid, Spain). The wing model was represented as a rigid flat plate with a thickness of 0.4 mm, and the wing edges were modeled as semicircles with a radius of 0.2 mm. In this simulation, the flexibility of the wings and the effects of the body were neglected [33]. The computational domain was defined as a cuboid (streamwise length = 600 mm, width and height = 300 mm) with the model positioned at its center. The coordinate system is aligned with the body coordinate system where the x-axis represents the wingspan direction, the z-axis represents the incoming flow direction, and the y-axis represents the upward vertical direction. The flow model is one-way external flow, reference length is the mean chord length of *A. dichotoma* with a Reynolds number (Re) set to 3400. The simulation is three-dimensional, and the turbulence model is LES. The wing motion in the simulation follows the kinematic patterns derived from the Euler angles of the *A. dichotoma* obtained through kinematic observations. A total of five flapping cycles were simulated with the fourth and fifth cycles, where the flow field was stable, selected for analysis. The streamwise length L1 was approximately 18 times the aerodynamic chord length of the model. The sidewall boundaries were set to periodic conditions, which served as an acceptable approximation for symmetric planes.

## 3. Results and Discussion

### 3.1. The Wing Extension and Folding Behavior of *A. dichotoma*

The entire sequence of *A. dichotoma’s* wing extension, flight, and folding process was captured through high-speed photography. Figure 2A illustrates the full wing extension process (including both elytra and hindwings), which took approximately 0.275 s. The wing extension process can be roughly divided into three stages. First stage, Figure 2A(a,b): the elytra first open laterally, then move upward and forward, rotating approximately 45°. Simultaneously, the hindwings begin to extend to the sides and move towards the abdomen, forming a 60° angle with the body. During this movement, the previously folded hindwings begin to unfold, although the wing tips remain folded. This phase lasts about 0.028 s. Second stage, Figure 2A(c,d): the elytra continue moving upward and forward until they reach their maximum elevation, rotating up to 112°, providing sufficient space for the hindwings to start flapping. The further opening of the elytra takes around 0.142 s. Third stage, Figure 2A(e–h): the not fully extended hindwings initiate the flapping motion, with the folded wing tips gradually unfolding during this process. After approximately 0.133 s of flapping, the hindwings are fully extended, transitioning the beetle into a flight state. When fully extended, the angle between the leading edge of the wings and the body is around 95°.

Figure 2B illustrates the complete wing retraction process (including both the elytra and hindwings) of *A. dichotoma*, which takes approximately 2.67 s. The wing retraction process can be roughly divided into three phases: The first stage (a,b) involves the gradual closing of the elytra while the hindwings remain extended. Second stage (c,d): the elytra continue to close and as they do, the hindwings gradually retract underneath them. During this phase, the hindwings begin folding, although the wingtip area remains unfolded. This closing process of the elytra takes about 0.426 s. The third stage (e–h) sees the elytra fully closed, covering most of the hindwings. Subsequently, a brushing motion, driven by the abdominal muscles in coordination with the elytra, fully folds the hindwings and tucks them beneath the elytra. This final phase lasts about 1.677 s.

The three-dimensional coordinates of the wing tips during the folding process were captured, revealing the motion trajectory of the wings, as shown in Figure 2D. The motion trajectories of the left and right wings during the folding process are generally symmetrical about the body’s longitudinal axis, presenting a “W” shape overall. The midpoint of the trajectory indicates the initial folded state when the left and right wings cross at the rear, with the flying wings pressed against the abdomen by the elytra. As the abdominal muscles contract, the wing tips move toward the head of the beetle, while the abdomen ascends due to muscular peristalsis, resulting in the upward movement of the wing tip trajectory. The time series indicates that most of the duration of the folding process is spent transitioning from the initial folded state to the fully folded state, primarily driven by the brushing motion induced by the contraction of the abdominal muscles.

Through multiple experimental video observations, it was found that during the second stage of the folding process, there may be instances where the left wing randomly covers the right wing, or vice versa, showing no significant difference between the two positions (*p* = 0.72, Kruskal–Wallis test). During the brushing motion, the hindwings do not fold under the elytra simultaneously; rather, one side is completely folded beneath the elytra before the other side begins to fold. The order of coverage is related to the sequence of folding; notably, when the hindwings are fully folded during the third stage, the hindwing that is covered beneath is always the first to fold.

**Figure 3:**
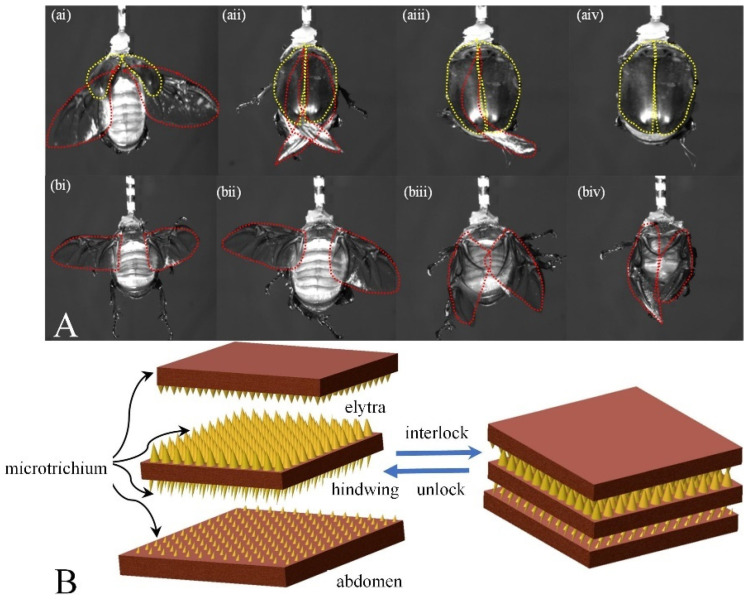
The locking mechanism of microtrichia and the effect of removing the elytra on the folding process. (**A**) The influence of the elytra on the wing-folding process. (**a**) The folding process with the elytra present; (**b**) the folding process after the elytra has been removed. (**B**) Schematic representation of the locking mechanism of microtrichia.

The inner surface of the elytra and the dorsal surface of the abdomen in *A. dichotoma* are equipped with microtrichia structures. These microtrichia play a crucial role in securing the folded hind wings by interlocking them through a brushing mechanism, preventing the folded wings from slipping out of the elytra. This brushing action is driven by the peristaltic movements of the abdominal muscles, which activate the dorsal microtrichia on the abdomen. During the folding process, the hindwing positioned below first comes into contact with the microtrichia on the dorsal side of the abdomen and is folded into the elytra under the guidance of abdominal muscle contractions [34]. At this point, the upper hindwing remains relatively fixed due to the locking effect of the microtrichia. Once the lower hindwing has completely folded, the presence of the microtrichia creates a self-locking mechanism between the elytra and hindwings as well as between the abdomen and hindwings, securing their positions, as illustrated in Figure 3B. Subsequently, as the abdominal muscles continue to contract, the upper hindwing is also folded and locked beneath the elytra with the aid of the microtrichia, completing the closure of both hindwings.

The majority of the elytra were removed, leaving only a small portion near the wing base, to observe the wing folding behavior, as shown in Figure 3A. Regardless of whether the elytra are present, both the first and second stages of wing folding occur. The main difference lies in the third stage. In beetles with intact elytra, during the third stage (Figure 3A(a)), the elytra compress the hindwings against the dorsal abdominal muscles. The inner surface of the elytra contains microtrichia, which cooperate with microtrichia present on the hindwings and the dorsal side of the abdomen. Through the peristaltic motion of the abdominal muscles, the brushing action gradually moves the wing tips toward the head of the beetle, allowing the wing tips to fully fold and retract beneath the elytra. Subsequently, the elytra descend to completely cover the hindwings, concluding the wing-folding process.

Conversely, when the beetle without elytra undergoes the third stage (Figure 3A(b)), the hindwings, controlled by the root muscles, initially converge towards the tip of abdomen. During this convergence, the hindwings do not contact the abdomen. This process does not require the cooperation of the elytra, allowing the hindwings to successfully converge and position their tips towards the tail. In the subsequent phase, the abdominal muscles begin to contract; however, due to the loss of the microtrichial cooperation from the elytra, the hindwings cannot be securely locked in place and also lose the downward pressure provided by the elytra. Although the abdominal muscles continue their peristaltic motion, the microtrichia on the hindwings struggle to engage with those on the dorsal side of the abdomen. Consequently, the brushing action fails to effectively transfer to the hindwings, preventing the wing tips from moving toward the beetle’s head as they would in a beetle with intact elytra, resulting in prolonged difficulty in completely folding the hindwings.

### 3.2. Wing Kinematics of *A. dichotoma* During Flight

**Figure 4:**
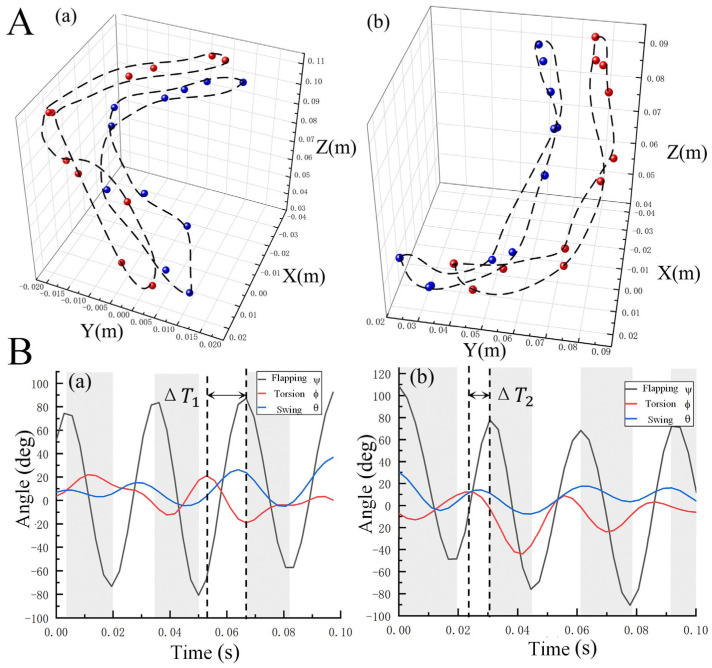
Wingtip trajectories and kinematic patterns of *A. dichotoma* during flight. (**A**) Flapping trajectories of two different individuals, represented in the inertial coordinate system by blue and red dots, respectively. (**a**) Left wing trajectory. (**b**) Right wing trajectory. (**B**) Kinematic patterns of wing motion for the two individuals. (**a**) The individual represented by the blue dot in (**A**). (**b**) The individual represented by the red dot in (**A**). The gray and white areas denote the downstroke and upstroke phases of the wings, respectively, with the phase difference between the torsion angle and flapping angle indicated by Δ*T*.

During steady flight, the flapping of the wings on both sides in insects is generally symmetrical [35]. The wingtip trajectories of the hindwings for five selected *A. dichotoma* samples were analyzed, with Figure 4A illustrating the three-dimensional trajectories of the hindwing tips during flapping for two representative individuals. As shown, the movements of the left and right hindwings are nearly symmetrical, exhibiting the same motion pattern and sharing a common stroke plane inclined at an angle of 60° to the horizontal. Upon examining the shape of the trajectories, an intersection point is observed near the inward rotation phase, revealing that the wingtip paths resemble a figure-eight pattern. This specific movement trajectory ensures the generation of significant lift, even with relatively small wing area.

The flapping motion of *A. dichotoma* is controlled by muscles at the wing root, and its kinematics can be described using the Euler angles *ψ*, *ϕ*, and *θ*. By processing and analyzing the data as shown in Figure 2C, we obtained the kinematic data for *A. dichotoma* at any given moment. Fast Fourier Transform (FFT) was applied to filter the raw data, resulting in the real-time curves of the three Euler angles during the flapping motion of two individuals, as depicted in Figure 3B. The trends in angle variation among different samples are largely consistent. The phases of the flapping and swinging angles are essentially the same, but there is a noticeable phase difference between the torsion angle and the other two angles (Δ*T*_1_, Δ*T*_2_), a phenomenon commonly observed in the flight of many insects, including butterflies and bamboo weevils [5,27,30]. In terms of amplitude variation, the flapping angle shows the greatest change, followed by the torsion angle, which has a slightly smaller amplitude, while the swinging angle exhibits the least variation. *A. dichotoma* achieves high lift through a significant flapping amplitude while instinctively adjusting its wing’s torsion angle to enhance flight efficiency, with the swing motion of the hindwings being relatively subtle [36,37,38].

To visually represent the temporal variations in the angles for subsequent analysis, a Fourier series was employed to further fit the calculated Euler angle data. In this study, a fourth-order Fourier series was utilized for fitting, with the Euler angles of wing motion defined as follows:

(3) $$\left\{\begin{array}{c} \psi \left(t\right)=a_{0}+\sum_{n=0}^{4}\left[a_{n}\cos\left(nKt\right)+b_{n}\sin\left(nKt\right)\right]\\ \phi \left(t\right)=c_{0}+\sum_{n=0}^{4}\left[c_{n}\cos\left(nKt\right)+d_{n}\sin\left(nKt\right)\right]\\ \theta \left(t\right)=e_{0}+\sum_{n=0}^{4}[e_{n}\cos\left(nKt\right)+h_{n}\sin\left(nKt\right)] \end{array}\right.$$

(4) $$K=\frac{2\pi fs_{1}}{2U_{w}}$$

(5) $$U_{w}=2\psi_{w}s_{0}f$$

*K* denotes the decay frequency, while *f* refers to the flapping frequency of the hind wings, with an average value of 32 Hz. *s*_1_ represents the average chord length of the wings, and *U*_w_ is the reference velocity at the wingtip. Additionally, *S*_0_ denotes the wingspan of 52 mm, and *ψ*_w_ indicates the flapping amplitude, measured at 158°. The Fourier coefficients *a*_n_~*h*_n_ are detailed in Table 2.

To further quantify the wing movement of the *A. dichotoma*’s flight, we defined the stroke amplitude angle Φ and the average stroke angle $\bar{\Phi }$. They are defined by $\Phi =(\alpha_{\max}-\alpha_{\min})$ and $\bar{\Phi }=(\alpha_{\max}+\alpha_{\min})/2$, where $\alpha_{\max}$ and $\alpha_{\min}$ are the maximum and minimum values of the Euler angle, respectively. These angles were calculated over six wingbeat cycles, which were selected from the latter half of the ten cycles recorded in the experimental groups. As shown in Table 3 and Table 4, the Φ of $\psi \left(t\right)$ ranged from 147.71° to 166.97°, with $\bar{\Phi }$ varying between −5.52° and 0.45°, resulting in a flapping amplitude of approximately 158°. The Φ of $\phi \left(t\right)$ varied from 31.44° to 56.16°, with $\bar{\Phi }$ ranging from −17.52° to 5.56°, indicating a torsion amplitude of about 40°. The Φ of $\theta \left(t\right)$ ranged from 12.32° to 31.70°, with $\bar{\Phi }$ changing between 0.04° and 8.39°, resulting in a swing angle amplitude of approximately 21°.

The flapping angle amplitude observed in this experiment closely resembles the angle reported by Nguyen et al. [14] in their observations of beetles during forward flight, although it is slightly less than the maximum flapping angle of 180° during hovering. The variation in the Euler angles is similar to the flight Euler angle patterns derived from the studies by Sun Mao et al. [15,39], where the Φ showed the greatest amplitude of variation, and the $\bar{\Phi }$ was very close to zero, slightly deviating in the negative direction from the coordinate axis. The variation in the torsion angle was smaller than that of the flapping angle, with the $\bar{\Phi }$ significantly deviating in the negative direction along the coordinate axis. In contrast, the swing angle showed a much smaller range of variation compared to the flapping and torsion angles. The $\bar{\Phi }$ of the swing angle remained near 0°, with an overall positive trend, indicating a consistent pattern throughout the experiment.

Tracking the wing motion of restrained insects has revealed that while wing restraint during flight affects the flapping force and reduces lift generation, it does not significantly alter the wingbeat trajectory [40,41]. Thus, it is feasible to extend the results obtained from restrained flight experiments to infer the wingbeat kinematics of free-flying insects. The slightly negative $\bar{\Phi }$ indicates that a greater portion of the wingbeat amplitude occurs below the horizontal plane. This suggests that during the downstroke phase, the beetle generates higher lift to counteract gravity and sustain flight throughout the wingbeat cycle. This phase plays a crucial role in lift-generation [42]. During restrained flight, the inclination of the stroke plane relative to the horizontal plane remains fixed at approximately 15°. The $\bar{\Phi }$ of the torsional angle deviates from the horizontal plane by 8°. This deviation is attributed to a slight elevation of the beetle’s body during wingbeats, which reduces the inclination of the stroke plane [43]. Throughout the wingbeat cycle, the wings undergo rapid twisting at the onset of the upstroke, maintaining a relatively large torsion angle during the upstroke. As the wings proceed into the downstroke, the torsion angle gradually decreases, generating a greater amount of lift.

### 3.3. Visualization of the Flow Field During Flight

**Figure 5:**
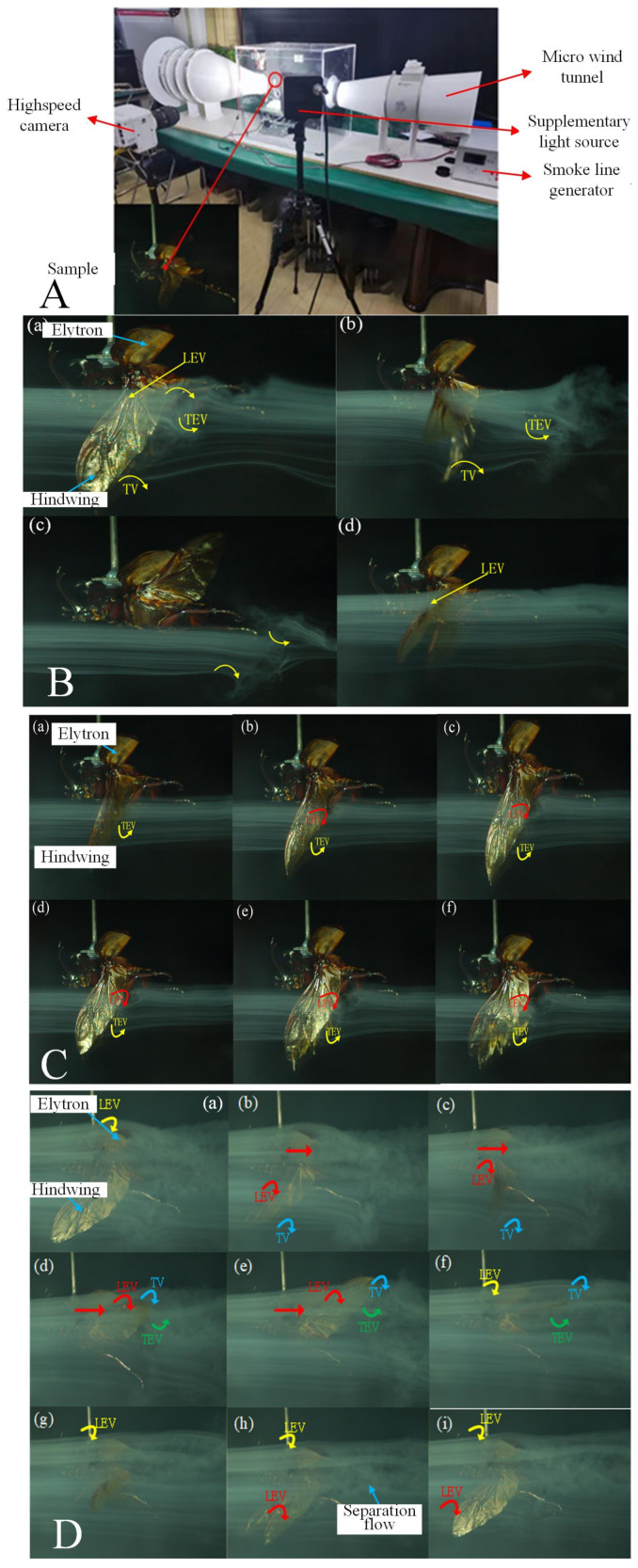
Visualization results of the flow field during the flight of *A. dichotoma*. (**A**) Flow field visualization experimental setup. (**B**) Flow field visualization for a flapping cycle. (**a**) Start of the upstroke, (**b**) upstroke process. (**c**) Start of the downstroke, (**d**) downstroke process. (**C**) Visualization of the flow field during the wing’s supination. (**D**) Flow field visualization illustrating the interaction between the elytra and hind wings.

During flight, *A. dichotoma* generates strong vortices around its body and wings, inducing lift [44,45]. To further investigate the aerodynamic characteristics of *A. dichotoma* in flight, flow field visualization experiments were conducted in a wind tunnel using a smoke line device (Figure 5A).

Figure 5B presents sequential images of the flow field visualization during the tethered flight of *A. dichotoma*, from the point of wing movement at its lowest position, marking the beginning of the upward stroke. The smoke plane is positioned 0.75 wing spans away from the wing root. It is observed that the smoke flow is deflected downward after passing over the beetle’s wings, with the deflected direction nearly perpendicular to the flapping plane (Figure 5B(a)). At the end of the downward stroke, the beetle enters a pronation phase, during which the hind wings undergo a twisting motion. Utilizing the Clap-and-Fling mechanism, the beetle induces a counterclockwise rotating Trailing Edge Vortex (TEV). As the inward rotation concludes and the upward stroke commences, both the counterclockwise TEV and the clockwise Tip Vortex (TV) are clearly visible in the images (Figure 5B(b)).

During the upward stroke, the vertical displacement of the smoke flow is minimal, indicating that the lift generated by the hind wings during this phase is limited. At the conclusion of the upstroke, the generated TV is captured during the outward rotation and moves backward relative to the body along with the downward stroke, where a significant separated flow is observed behind the body. During the downstroke, a leading-edge vortex (LEV) is generated on the hind wings, gradually increasing in size as the hind wings move downward. This vortex attaches to the hindwings, contributing to lift. *A. dichotoma* exhibits significant lift during the downward stroke, akin to other insects that flap their wings with angled stroke plane.

*A. dichotoma’s* wing rotation process is closely observed, as illustrated in Figure 5C. The smoke plane is positioned 0.25 wing spans away. Initially, the leading edges of the hind wings are close together; as the wings undergo twisting, the leading edges move apart while the trailing edges come closer together, inducing a counterclockwise rotating TEV. This twisting motion forces the smoke flow to rapidly pass over the open leading edges, becoming entrained in the gap between the two hind wings, as shown by the curved smoke lines in Figure 5C(d) that flow around the hind wings. Simultaneously, the hind wings perform rapid opening and twisting movements in a very short time, resulting in the formation of a LEV on the hind wings. This vortex attaches to the hind wings, following their motion and continually growing, thereby providing lift for the beetle during flight.

The flow field visualization results reveal the wing–wing interaction between the elytra and the hindwings. The elytra move in a stroke plane that is nearly vertical relative to the body, while the hindwings move upward and backward within an inclined stroke plane. Structurally, the elytra are positioned above the hindwings, which appears to disrupt the coherent flow of the LEV on the hindwings. As the smoke flow approaches the gap between the elytra and hindwings, it is rapidly drawn in and then accelerates outward, leading to deflection after passing over the beetle’s body, as observed in Figure 5D(b–e). During the upstroke, the induced airflow moves over the leading edge of the hindwings, while the hindwings continue to move upward. This process enhances the size of the LEV attached to the hindwings due to the suction effect, resulting in increased lift for the beetle [46].

At the beginning of the downstroke, both the elytra and hindwings flap simultaneously, generating a relatively small LEV above the elytra. Due to the high curvature and limited stroke amplitude of the elytra, the LEV remains attached to their surface throughout the downward motion. Toward the end of the downstroke, the hindwings move rapidly, creating accelerated airflow that causes the size of the LEV on the elytra to continuously increase, remaining observable until the start of the upstroke. However, during the flapping cycle, separation flow can be observed on the hindwings but not on the elytra, indicating that the lift generated solely by the elytra contributes minimally to the total lift. Instead, the elytra play a more significant role in enhancing the lift of the hindwings through wing–wing interactions [47].

**Figure 6:**
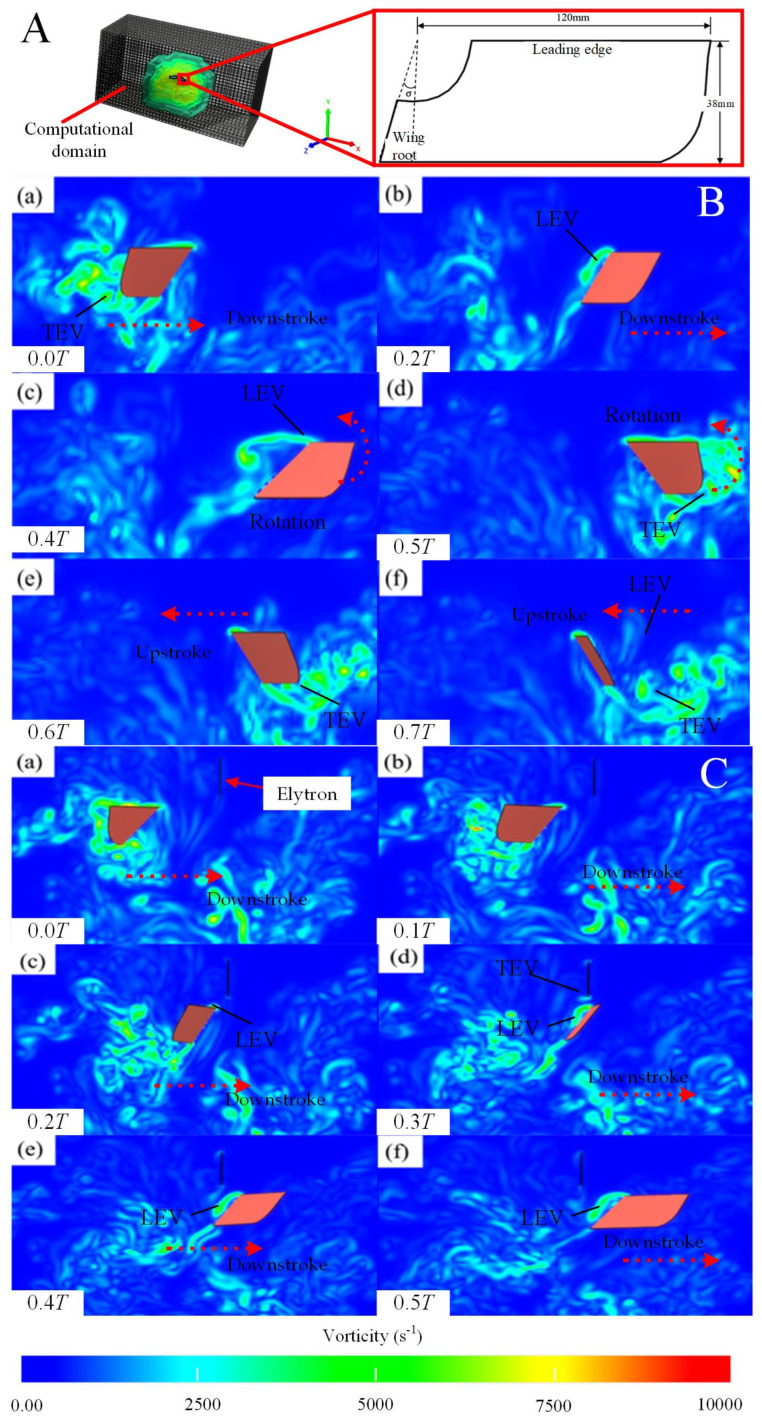
Vorticity contours of the flapping motion of the *A. dichotoma* wing. (**A**) Calculation domain and simulation model wing schematic. σ is the angle between the wing root and the vertical direction. (**B**) Vorticity contour for a single flapping cycle without elytron. Images (**a**–**c**) represent the vorticity during the downstroke phase, while images (**d**–**f**) depict the vorticity during the upstroke phase of the flapping cycle. (**C**) Vorticity contour for downstroke stage with elytron. In both panels, the red dotted line represents the direction of the wings’ movement, and the distinct LEV and TEV are identified.

For the CFD simulation results, a slice plane was created 50 mm from the wing root, parallel to the Y-Z plane of the body coordinate system, to capture the vorticity contours of the beetle’s flapping motion during a single flapping cycle, as shown in Figure 6A. In Figure 6B(a), the initial state reveals a TEV generated from the previous flapping cycle, which gradually sheds as the wing moves forward. In Figure 6B(b), the new LEV generated during the current downstroke is visible, slightly detached from the wing surface and extending along the chordwise direction across the upper surface. The TEV forms due to the interaction between the trailing edges of the left and right wings and is clearly observed after wing separation in Figure 6B(e). Figure 6B(d–f) illustrate the transition of the wing into the upstroke phase, where the former lower surface flips to become the new upper surface, generating a new LEV. As the upstroke progresses, the LEV gradually detaches until the next cycle generates a new vortex. Simultaneously, the TEV sheds rapidly, merging with the wake left by the wing during the flip. Throughout the entire flapping cycle, the LEV remains closely associated with the wing, which is one of the key mechanisms enabling the beetle to generate sufficient lift.

Next, the elytron model was imported into the software and positioned directly above the left and right wings, with a fixed angle of attack set to 90°, maintaining a static position. The resulting vorticity contours during flapping with elytron are shown in Figure 6C. The panels of Figure 6C(a–c) depict the vorticity fields when the wing approaches the elytron, while Figure 6C(d–f) show the vorticity fields when the wing moves away from the elytron. As the wing nears the elytron, the leading-edge vortex (LEV) is amplified, delaying vortex shedding and causing the LEV to remain attached to the wing surface until the end of the downstroke, thus maintaining lift. During this phase, as the hindwing approaches, a LEV and a trailing-edge vortex (TEV) also form on the elytron, which then mix with the wing’s LEV and shed, as seen in Figure 6C(e). The TEV generated on the wing rapidly detaches as the wing twists at the end of the downstroke, creating a complex flow field around the wing, as shown in (f). Therefore, the presence of the elytron enhances lift by generating additional vortices and delaying the dissipation of the LEV.

## 4. Conclusions

This study utilized a motion capture system to conduct a detailed investigation into the flight behavior and kinematics of *Allomyrina dichotoma*. The findings reveal that the wing-folding process in this beetle is primarily driven by thoracic and abdominal muscles and coordinated through the interactions between the elytra, hind wings, and dorsal abdominal microstructures. The primary role of the elytra is to secure and lock the hind wings, apply downward pressure to facilitate their close alignment with the abdomen, and create a chamber-like environment conducive to wing folding.

Using Euler angle definitions and Fourier series, we precisely described the instantaneous motion patterns of the beetle across multiple flapping cycles. Flow visualization revealed the generation and evolution of vortices on both the hind wings and elytra, providing initial insights into the mechanisms underlying the beetle’s high lift production. By combining quantitative kinematic data with CFD simulations, we further verified the contribution of elytra–wing interactions to overall lift enhancement.

This work offers a detailed observation and quantification of *A. dichotoma’s* flight behavior, providing accurately obtained kinematic data that will serve as a valuable reference for future studies of insect flight. Furthermore, the coupling mechanisms between the hind wings and elytra may be applied to the development of flapping-wing micro air vehicles (FMAVs), with potential benefits in increasing lift, optimizing aerodynamic efficiency, and protecting wing structures.

## Figures and Tables

**Table 1:** Morphological data of *A. dichotoma*.

Sample	1 (F)	2 (M)	3 (F)	4 (M)	5 (M)	Mean (Standard Deviation)
$M_{1}$ (g)	6.35	6.84	7.54	6.69	6.76	6.84 (0.39)
$M_{2}$ (mg)	46.31	55.44	61.32	49.19	49.63	52.36 (5.35)
$M_{3}$ (mg)	56.27	54.07	65.63	61.61	60.10	59.53 (4.04)
$s_{0}$ (mm)	49.35	53.31	56.50	50.76	51.28	52.24 (2.48)
$s_{1}$ (mm)	16.34	17.58	19.87	18.67	18.93	18.27 (1.21)
$l_{0}$ (mm)	62.45	65.34	69.52	64.98	63.27	65.1 (2.44)
$l_{1}$ (mm)	18.36	18.81	19.21	18.56	18.12	18.61 (0.37)
$s_{0}/s_{1}$	3.02	3.03	2.84	2.72	2.71	2.87 (0.21)
$l_{0}/l_{1}$	3.40	3.47	3.62	3.50	3.49	3.50 (0.07)
$A_{w}$ (${\mathrm{mm}}^{2}$)	806.40	937.19	1122.65	947.69	790.75	920.94 (88.63)
γ (mg.${\mathrm{mm}}^{-2}$)	0.06	0.06	0.05	0.05	0.06	0.06 (0.01)

**Table 2:** Constant coefficients of the Fourier series of *A. dichotoma*.

	$a_{0}$	$a_{1}$	$a_{2}$	$a_{3}$	$a_{4}$	$b_{1}$	$b_{2}$	$b_{3}$	$b_{4}$
$\psi_{1}\left(t\right)\psi_{1}\left(t\right)$	4.16	−5.25	5.95	−7.78	10.85	−3.73	0.47	−6.09	−16.18
$\phi_{1}\left(t\right)$	−0.27	3.25	3.39	−7.25	8.35	3.11	1.60	6.75	0.99
$\theta_{1}\left(t\right)$	−7.74	0.47	−1.43	−1.04	−10.10	6.46	1.76	−4.78	−1.26
$\psi_{2}\left(t\right)$	1.17	4.16	1.80	−1.00	38.37	0.37	1.04	2.60	5.83
$\phi_{2}\left(t\right)$	0.61	−5.49	3.09	−2.97	0.72	−10.50	0.36	−0.72	6.92
$\theta_{2}\left(t\right)$	7.78	2.00	1.32	1.88	9.29	−1.20	2.02	−3.90	−4.10

**Table 3:** Stroke amplitude angle Φ of *A. dichotoma*’s wing movement.

Φ (°)	$\psi \left(t\right)$	$\phi \left(t\right)$	$\theta \left(t\right)$
Cycle 1	160.37 (17.21)	31.44 (8.56)	31.70 (2.85)
Cycle 2	158.79 (15.52)	52.87 (12.73)	25.19 (16.63)
Cycle 3	153.83 (21.03)	56.16 (2.67)	21.85 (6.73)
Cycle 4	147.71 (11.77)	33.57 (18.62)	12.32 (8.64)
Cycle 5	162.62 (18.92)	26.93 (22.21)	15.12 (3.79)
Cycle 6	166.97 (16.64)	39.12 (16.45)	22.08 (13.61)

**Table 4:** Average stroke angle $\bar{\Phi }$ of *A. dichotoma*’s wing movement.

$\bar{\Phi }$ (°)	$\psi \left(t\right)$	$\phi \left(t\right)$	$\theta \left(t\right)$
Cycle 1	−2.20 (10.67)	5.56 (6.89)	0.04 (8.82)
Cycle 2	−5.53 (2.85)	−17.53 (17.52)	4.89 (11.62)
Cycle 3	0.45 (12.53)	−15.88 (15.43)	3.22 (6.48)
Cycle 4	0.28 (11.75)	5.34 (11.73)	2.56 (7.83)
Cycle 5	−4.57 (21.91)	−10.35 (9.68)	8.39 (15.84)
Cycle 6	−3.78 (13.65)	−16.53 (12.85)	6.59 (14.49)

## Data Availability

The data presented in this study are available upon request from the corresponding author.
